# Development of a Social Communication Questionnaire (QSC-ID) for People With Intellectual Disability in a Deaf Sample: A Pilot and Feasibility Study

**DOI:** 10.3389/fpsyt.2021.755993

**Published:** 2021-12-08

**Authors:** Chantal Weber, Christoph Weber, Johannes Fellinger, Daniel Holzinger

**Affiliations:** ^1^Institute of Neurology of Senses and Language, Hospital of St. John of God, Linz, Austria; ^2^Research Institute for Developmental Medicine, Johannes Kepler University Linz, Linz, Austria; ^3^Department for Inclusive Education, University of Education Upper Austria, Linz, Austria; ^4^Division of Social Psychiatry, Medical University of Vienna, Vienna, Austria; ^5^Institute of Linguistics, University of Graz, Graz, Austria

**Keywords:** social communication, intellectual disability, deafness, hearing loss, caregiver questionnaire

## Abstract

**Background:** Social communication (SC) includes the use and interpretation of verbal and non-verbal messages within a social context and thus requires more than knowledge of language. Social communication skills are essential for connecting and engaging with others, and SC deficits are often associated with emotional and behavioral problems. There is a lack of feasible instruments for assessing SC skills in individuals with intellectual disability (ID).

**Methods:** A questionnaire on social communication in adults with ID (QSC-ID) comprising 20 Likert-scaled items was developed and completed on behalf of participants (*n* = 52) from three Austrian therapeutic living communities for people with ID and deafness by their living- and working-facility key caregivers. The sample of adults with hearing loss was considered ideal for the development of a measure of SC that is not restricted to a specific communication mode or overly related with language skills.

**Results:** The preliminary results showed high construct validity. Correlations were high between SC and language, social skills, and severity of autism spectrum disorder (ASD), moderate between SC and adaptive skills, and non-verbal intelligence and, as expected, low between SC and motor skills. Interrater reliability was found to be good or at least acceptable for all items. Total raw scores were well-distributed over the whole range—Cut-offs based on the 10th and 20th percentile are suggested to identify atypical and borderline SC skills. Caregiver feedback and completeness of data suggest that the questionnaire is highly feasible.

**Conclusion:** Questionnaire on social communication in adults with ID is an easy-to-use caregiver-reported questionnaire for use with individuals with mild to severe forms of ID. Initial testing of validity looks promising. Further validation in populations with typical hearing is required. Due to substantial correlations between SC and structural language skills the calculation of specific SC cut-offs for different levels of linguistic skills should be considered when sufficient data is available.

## Introduction

Social communication (SC) can be defined as the appropriate use and interpretation of verbal and non-verbal messages within a social context and thus includes much more than the knowledge of language (e.g., vocabulary and grammatical rules). It involves the competence of using language as a “means of connecting and engaging with others” (1) including three major skills described by the American Speech-Language-Hearing Association (2). Firstly, SC serves a rich variety of communicative functions of varying degrees of complexity, ranging from attracting somebody's attention to expressing requests and more sophisticated uses of language (e.g., giving hints and using language to persuade others). Secondly, SC includes the mastering of reciprocity and following the rules of conversation (including turn-taking skills, topic adherence, and communicative repair strategies). Thirdly, another major skill involved is the adaptation of verbal or non-verbal communicative behavior in response to the interlocutor or situation (e.g., use of varying degrees of politeness or adapting communication content to the presumed interests and prior knowledge of the interlocutor). The terms “social communication” and “pragmatics” are often used interchangeably. Social communication has a more functional definition and must be considered as a much broader concept that, unlike pragmatics, includes non-verbal communication (3).

Social communication skills are associated with multiple dimensions of human development, particularly with language skills and here especially with the ability to understand words and sentences. The majority of studies included in the review by Matthews et al. (4) showed significant correlations between formal language and pragmatic language ability. The role of social interaction in language development has also been emphasized in the theoretical models of language acquisition by Bruner (5) and Tomasello (6), and demonstrated empirically by Gilkerson et al. (7). Significant correlations between SC/pragmatic and language skills have been described in total population cohorts (8) and in specific populations, such as adolescents with behavioral problems (9) and children with language impairment (10). Acquisition of SC skills involves the dynamic interaction of language, social, and cognitive development (3) including the socially appropriate use of language that primary requires social cognition (e.g., theory of mind). In addition, associations between SC ability and a number of other cognitive abilities such as cognitive flexibility (11), executive function (4), and reasoning ability (12) are empirically well-documented. Martin et al. (13) described moderate correlations with non-verbal cognition. Daily living skills include both practical skills and skills required for community living, and are partly acquired through SC with peers or care-givers.

Intellectual disability (ID) is a developmental condition defined by deficits in intellectual functioning, adaptive skills, social behavior, and language and communication (14). As SC competence requires the complex integration of language, social, and non-verbal cognitive skills, SC is usually affected in individuals with ID (13, 15). Between 35 and 46% of people with ID have a co-occurring hearing loss (16), and ID has been described in 13–21% of individuals who are deaf or hard of hearing (DHH) (17, 18). The dual diagnosis of ID and deafness is usually linked with specific challenges in communication. Depending on the degree of hearing loss, signed language (often a variety of a national sign language with a simplified grammatical and lexical system or simultaneous communication of spoken and signed language) is the preferred means of communication for many of those who are DHH and have ID. In addition, most of them have experienced severe communicative and social deprivation since early life. The great majority have grown up in families with typical hearing with little or no systematic visual communication. Their limited participation in a rich variety of everyday SC situations and the impossibility of overhearing communication between others impacts not only language development, but also the development of SC skills (19). Despite the recent advances in language development observed in children with pre-lingual deafness due to universal newborn hearing screening, early fitting of hearing aids and/or cochlear implantation, and access to modern family-centered early intervention including the availability of signed language (1) SC (pragmatic) difficulties are still regarded as a serious challenge in the new generation of children who are DHH with or without an additional ID. Yoshinaga-Itano et al. (20) identified ID as one of the risk factors for SC development in the new generation of children who are DHH. Other risk factors were delayed identification and intervention, limited family communication, and higher degrees of hearing loss, that are true for most of today's generation of adults who are DHH and have ID.

While the need for access to visual communication for individuals who are DHH and have ID is increasingly recognized, the outstanding role of SC in the development of language, learning, social relationships, and mental health is often overlooked. Social communication difficulties in children who are DHH have been associated with higher rates of behavioral problems (21, 22). Similarly, Helland et al. (9) reported stable associations between pragmatic language problems and behavioral problems in children with language impairment. In a representative longitudinal total population study (Avon Longitudinal Study of Parents and Children), Law et al. (23) demonstrated the role of children's SC skills at the age of 8 years as a mediator between early family-related social risks and mental-health outcomes (emotional and conduct problems, hyperactivity, and peer problems) at age 13. Accordingly, disproportional problems in the social use of language compared to language knowledge have been found to be associated with mental health problems, such as conduct problems and hyperactivity, and with problems with peers (24). Finally, Whitehouse et al. (25) described adverse outcomes in adult life, particularly problems establishing friendships, in children with pragmatic language difficulties.

Despite the prominent role of SC in many domains of human development, its broad neglect in conceptualizations of intervention programs, in the design of working and living environments for people with ID and in individualized intervention planning might, to some extent, be a consequence of the lack of feasible measures of SC skills or deficits. Even though an adaption and validation of the social communication questionnaire for adults with intellectual disability (SCQ-AID) (26) is available, it has primarily been constructed to identify symptoms of autism spectrum disorder (ASD). Accordingly, items of the SCQ-AID relate to social relations, special interests, and specific communicative behaviors observed in individuals with ID. Similarly, the pervasive developmental disorder in mental retardation scale (PDD-MRS) (27) and the diagnostic behavioral assessment for ASD-revised (DiBAS-R) (28) screen for the presence or absence of autistic behaviors in adults with ID (29, 30). Other self-completion and face-to-face computer-assisted interviews have been used to collect data about SC abilities in people with ID (31). To our knowledge, the SCQ-AID is the only measure relating to SC (amongst other behaviors) that has been validated for adults with ID. Although there is quite a robust literature now on the SC profiles of DHH children [see systematic review by Crowe and Dammeyer (32)], we do not know of any validated measures of SC skills with ID who are DHH.

The objective of our observational pilot study was therefore to develop and evaluate an easy-to-use questionnaire for assessing SC in people with ID that is independent of communication modality (signing or speaking communication setting) and specifically focused on the social use of verbal and non-verbal messages in conversational encounters rather than structural language skills. Therefore, the population of adults with ID and hearing loss with their considerably heterogeneous cognitive skills, most often delayed social, linguistic, and social communicative skills and use of diverse communication modalities was considered an ideal sample for the development of a questionnaire on SC that could be of general use for individuals with ID. In addition to feasibility (particularly time efficiency of completion, scoring of the instrument, and acceptance by caregivers), the test quality of the new instrument (internal consistency, interrater reliability, and construct validity) was evaluated. This pilot study formed part of a comprehensive research program focusing on the needs of individuals who are DHH and have ID.

## Materials and Methods

### Construction of the Instrument

The questionnaire on social communication in adults with ID (QSC-ID) (Table 1) is intended to collect data on five aspects of SC that are assumed to be strongly interrelated: (i) an individual's engagement in SC, (ii) conversational skills, (iii) adaptation of communicative behaviors to an interlocutor, (iv) pro-social use of communication, and (v) use of non-verbal communication. The instrument is designed for use with individuals living and/or working in assisted communities who are at least minimally verbal (defined by a minimum of 25 expressive signs/words), and is independent of language or communication mode (e.g., spoken or signed). The QSC-ID is a proxy measure to be used by caregivers in assisted living and working facilities to collect information on SC skills that need to be considered in planning interventions, be they related to the adaptation of the caregiver's communication to an individual's needs, the promotion of an individual's SC skills in everyday life or in therapeutic settings, or adaptations of the environment. Therefore, the measure must be highly feasible (accepted by caregivers in terms of comprehensibility, time economy, and simplicity of scoring).

**Table 1:** Questionnaire social communication: QSC-ID (Translation from the German original).

**Questionnaire social communication QSC-ID**		**Applies fully**	**Mostly**	**Partly**	**Slightly**	**Applies not at all**
1.	Starts a conversation by himself/herself (shows communicative initiative).					
2.	Enjoys communicating with other clients/peers.					
3.	Enjoys communication with caregivers.					
4.	Enjoys communication with strangers.					
5.	Communicates with many different clients/peers.					
6.	Can stay in a longer two-way conversation (over 5 min).					
7.	Adheres to conversational rules (e.g., can wait for his/her turn).					
8.	Picks up on the interlocutor's contribution to the conversation and responds to it.					
9.	Stays on topic with his/her contributions to the conversation.					
10.	Tells others things they already know or don't care about.					
11.	Asks when he/she does not understand.					
12.	Can conduct a balanced conversation (send–receive).					
13.	Participates in group conversations with other clients/peers.					
14.	Involves himself/herself actively in group conversations.					
15.	Asks specific questions in group conversations.					
16.	Enjoys expressing himself/herself in front of a group.					
17.	Gives other clients/peers positive feedback/compliments and expresses appreciation through signed/spoken communication.					
18.	Communicates the offer of assistance to other clients/peers.					
19.	Uses eye contact and facial expressions appropriately.					
20.	Shows an appropriate sense of humor.					

Nine (of 20) items refer to an individual's *engagement in SC* in increasingly socially demanding situations that extend from communication with caregivers (item 3), specific peers (item 2), a variety of peers (item 5), communication in group contexts (items 13, 16) to communication with strangers (item 4). Furthermore, the individual's initiative (active engagement) in communication is elicited (items 1, 14, and 15).

Five questions target a person's *conversation skills*: the maximum length of two-way conversations possible (item 6), adherence to conversational rules (waiting for one's turn) (item 7), topic adherence (item 9), use of simple repair strategies (item 11), and the balance between sending and receiving messages (item 12).

A group of three items (items 8, 10, and 20) refers to the *adaptation of communication to the interlocutor* that requires the ability to attribute mental states such as knowledge, intentions, or beliefs (theory of mind) to another person.

Two items (17, 18) target *pro-social communication skills* (e.g., praise, compliments) that are assumed to support social integration within assisted communities. Finally (item 19), appropriate use of *non-verbal communication* is elicited. The QSC-ID provides a five-step Likert scale from “applies fully” to “does not apply at all.”

### Additional Measures and Adaptations

Table 2 shows additional measures used to collect supplementary data between 2017 and 2018 that provide information about the participants' profiles and were used to examine correlations between SC and individual developmental domains during first stages of validation.

**Table 2:** Additional measures and adaptations.

**Non-verbal cognition**
-*SON-R 6–40, SON-R 2 ½−7*
**Adaptive skills**
-*Vineland-II (Vineland adaptive behavior scales)*
**Language skills**
-*Adaption of RDLS-III (Reynell developmental language scale III)*
-*PMLP (Adapted scale for evaluating expressive skills based on the profile of multiple language proficiency)*
**Symptoms of Autism spectrum disorder**
-*ADOS-2 (Adapted version of the autism diagnostic observation schedule-2)*
-*DiBAS-R (The diagnostic behavioral assessment for autism spectrum disorders-revised)*
**Feasibility**
-*Completeness of data, caregiver feedback*

#### Non-verbal Intelligence and Adaptive Skills

For non-verbal intelligence testing, SON-R 6–40 (33) was used with most of the participants. SON-R 2 ½−7 (34) was used with participants with a non-verbal cognitive functioning level below 6 years. Adaptive skills, including personal and social skills, needed for everyday life were rated on the Vineland Adaptive Behavior Scales [Vineland-II (35)], a standardized assessment tool that utilizes semi-structured interviews to measure adaptive behavior. The Vineland Scales consist of various subscales, of which the following were of primary interest to our pilot study: (i) social skills, (ii) daily living skills, and (iii) motor skills (fine and gross).

#### Language Skills

Given the lack of instruments tailored to people with ID using primarily visual communication, two standardized assessments for spoken language were adapted for use in this pilot study. The language comprehension scale of the Reynell developmental language scale-III (RDLS-III) (36), originally developed for spoken English and normed for the age range of 1.10–6.11 years, was adapted to Austrian Sign Language by a group of hearing and deaf experts from the fields of linguistics, speech therapy, and pedagogy (37). The RDLS-III adaptation measures sign language comprehension of single signs and increasingly grammatically complex directions and statements. It consists of 62 items, including 15 single signs and 47 sign-language utterances presented in the context of acting out and pointing at pictures (play materials and four-field tables). The adaptation has so far been initially validated only with a small sample of 10 children born to deaf parents (aged between 1.10 and 9.7 years) growing up with Austrian Sign Language as their first language. This study demonstrated high correlations between the raw scores and participant age as well as non-verbal cognitive age. Furthermore, high correlation with parental assessments of language comprehension was found. The measure is therefore regarded as promising for use in the assessment of language comprehension in signing populations (37).

The eight-level scale of the profile of multiple language proficiencies (PMLP) (38) is used by caregivers to approximate expressive language skills. Originally developed for American Sign Language, and especially for children using multiple communication modalities, it was adapted to Austrian Sign Language by one of the authors [DH].

#### Autism Spectrum Disorder

Two different instruments were used for the assessment of ASD within the sample: Firstly, The autism diagnostic observation schedule (ADOS-2) (39) was adapted for use with adults with ID and deafness (40). It includes a number of age-appropriate play-based activities that are designed to obtain information in the areas of communication, reciprocal social interactions, and restricted and repetitive behaviors associated with a diagnosis of ASD. Secondly, the DiBAS-R (28) was used for ASD assessment. Diagnostic behavioral assessment for ASD-revised is a highly feasible psychometrically sound scale for screening for ASD in adults with ID. Nineteen questions are based on the current diagnostic criteria for early childhood or atypical autism (ICD-10: F84.0/F84.1) and ASD (DSM-5: 299.00) and refer to interactional, communicative, and sensory abnormalities and repetitive and stereotypical behaviors. The screening instrument can be administered by a caregiver or support person with no specific knowledge of ASD.

#### Feasibility

Three measures were used to evaluate the feasibility of the QSC-ID: (i) completeness of data, (ii) response rate, and (iii) caregiver feedback. A short feedback form focusing on acceptability and practicality of the questionnaire was developed based on the recommendations for feasibility studies by Bowen et al. (41). It includes four questions on (i) time efficiency (i.e., time for completing the questionnaire), (ii) individual effort (i.e., whether completing the questionnaire was easy, acceptable, or difficult), (iii) comprehension of the questions and examples, and (iv) estimation of usefulness of the QSC-ID in assessing a client's SC skills.

### Participants

The participants lived and/or worked in one of three therapeutic living communities (Lebenswelt) in Austria, two of which are located in Upper Austria and one in Lower Austria. The first Lebenswelt community for people with deafness and ID was established by the Hospital Barmherzige Brüder Linz in 1999 (42). Lebenswelt is characterized by a developmentally sensitive milieu therapy program that was developed specifically to cater to the needs of people with deafness and additional disabilities, focusing on visual communication and social relationships through individualized treatment and support programs (43, 44). Promoting SC skills in all contexts of life is a primary aim of Lebenswelt. Staff, whether deaf or hearing, are continually trained in sensitive strategies to support the residents in their development of language and SC.

**Figure 1:**
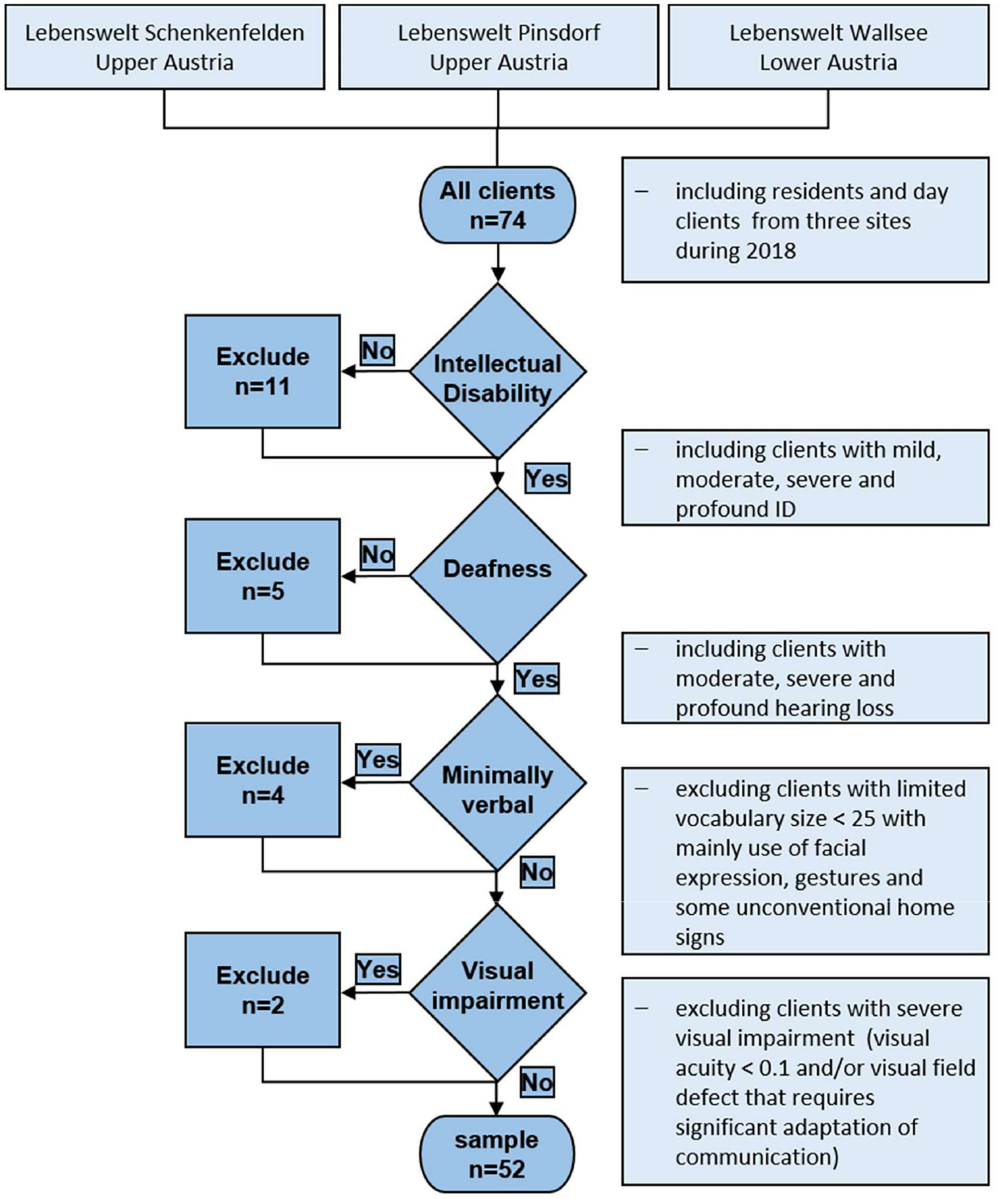
Participant recruitment flow chart.

The residents of Lebenswelt are a very heterogeneous group of individuals who are deaf/hard-of-hearing and have additional disabilities, especially in terms of their language and communication profiles. Our intention was to develop and evaluate an instrument that is independent of communication mode and can be used with a large variety of adults with ID. The sample inclusion criteria for this pilot study were therefore made as broad as possible, including participants with cerebral palsy, emotional and severe behavioral disorder, ASD, and/or other psychiatric disorders. Individuals with a non-verbal IQ higher than 69 (*n* = 11) or a pure tone threshold on the better ear above 40 dB (*n* = 5) were excluded from the sample. Non-verbal or minimally verbal individuals (*n* = 4) with a limited expressive vocabulary size <25, corresponding to level 1 of the adapted PMLP, were also excluded from the sample. Presence of a severe visual impairment and/or a visual field defect that requires significant adaptation of communication led to the exclusion of two more residents (Figure 1).

The final sample included 52 participants: 38 residents and 14 non-residents from all three Lebenswelt sites. All participants and their legal guardians (where applicable) gave permission for inclusion in our pilot study of anonymized participant data collected as part of the therapeutic program. The pilot study was approved by the ethics commission “Barmherzige Schwestern und Barmherzige Brüder” (EKB 14/18; 14.01.2019).

Sample characteristics are presented in Table 3. The majority (64.0%) of participants were male. Half of the sample had an ID in the severe (to profound) range with a non-verbal cognitive age below 6 years, and the remaining sample in the moderate and mild range (above 6 years). All participants except one with moderate hearing loss in one ear were severely to profoundly deaf, and 7 (14.0%) had been diagnosed with ASD. For rates of neurological comorbidities (epilepsy and cerebral palsy), see Table 3.

**Table 3:** Participant characteristics (*n* = 52).

	**Descriptives**
Age *M* (*SD*)	45.88 (19.64)
Sex male *n* (%)	33 (64.0)
Non-verbal cognitive functioning reference age (%)	
*>6 years*	23 (46.0)
*3–6 years*	26 (52.0)
* <3 years*	1 (2.0)
Degree of hearing loss *n* (%)	
*Moderate hearing loss (40–69 dB)*	1 (2.0)
*Severe hearing loss (70–89 dB)*	7 (14.0)
*Profound hearing loss (>89 dB)*	44 (85.0)
Autism spectrum disorder *n* (%)	7 (14.0)
Epilepsy *n* (%)	13 (25.0)
Cerebral palsy *n* (%)	16 (31.0)

### Procedure

For each participant, the QSC-ID was completed by two key caregivers, one of whom was responsible for the participant at work and the other in the group homes. Thus, for Lebenswelt residents each questionnaire was intended to be completed by the key caregiver in the working facilities (CW) and the key caregiver in the living facilities (CL) (*n* = 38). For individuals living at home or in another institution who came to Lebenswelt exclusively to work only one caregiver (CW) completed the questionnaires (*n* = 14). For four participants no CL report was available. With four questionnaires returns missing, the data from 86 of 90 tests were used for the calculations presented.

All IQ tests (SON-R 6–40, SON-R 2 ½−7) were administered and scored between 2017 and 2018 by a clinical psychologist with high sign-language competence. All tests were carried out in a calm, familiar environment on the Lebenswelt sites. In 2018, The Vineland-II Adaptive Behavior Scales were rated for each participant by a caregiver interviewed by a clinical psychologist, both of whom knew the clients very well. Scored by the clinical psychologist, the outcomes were used primarily to derive individualized treatment and support strategies for the clients' daily lives.

In 2018, as part of the present pilot study, the adapted RDLS-III language comprehension scale was used with each participant by one of the authors [ChaW], who has a high level of sign-language proficiency. The language assessments were carried out in a calm, familiar environment on the Lebenswelt sites.

The Austrian Sign Language adaptation of the PMLP (38) is regularly used by caregivers in the Lebenswelt therapeutic communities to guide intervention planning. The scale is part of a detailed communication observation sheet, and between 2017 and 2018 it was completed for each participant by the key caregivers.

Every participant underwent an in-depth multi-disciplinary diagnostic assessment for ASD during data collection. Firstly, this involved administration of the adapted ADOS-2 by a multidisciplinary team competent in signed communication and with many years of clinical experience in the fields of hearing loss, ID and ASD over the whole life course (40). Secondly, DiBAS-R with minimal adaptations to signed communication was completed by a clinical professional at the therapeutic living communities who had insights into the daily behaviors of each participant during the period of data collection.

Caregivers and members of the Lebenswelt therapeutic communities were involved in a meaningful way in the initial testing and evaluation of the questionnaire QSC-ID. After QSC-ID completion, all caregivers involved were asked to give their feedback on the feasibility of the questionnaire procedure.

### Statistical Analysis

In the first step, we conducted an exploratory factor analysis (EFA) with principal axis factoring, on CL and CW reports separately, to assess the dimensionality of the QSC-ID. Given high factor loadings, a low number of factors, and a high number of items, EFA can yield reliable results even for very small samples (*n* <50), as demonstrated by de Winter et al. (45). To assess for the reliability of the QSC-ID, we estimated Cronbach's Alpha and item-scale correlations (i.e., the correlation between a single item and the total score per factor after exclusion of the item) to judge internal consistency. Further, we estimated interrater reliability between CW and CL. We report intraclass correlations (ICCs) for consistency between two raters based on a two-way mixed model (46). Intraclass correlations were judged as follows [see Koo and Li (46)]: <0.50 = poor, 0.50–0.75 = moderate, 0.76–0.90 = good, >0.90 = excellent. Finally, to provide evidence of convergent and discriminant validity, we correlated the QSC-ID to several other measures. Convergent validity refers to how closely the new scale is related to other measures of a similar or the same construct. Discriminant validity is shown by a lack of correlations with unrelated constructs. Finally, for the interpretation of QSC-ID scores cut-off points were suggested. In accordance with procedures used for other instruments [e.g., Strengths and Difficulties Questionnaire, (47)], QSC-ID scores below the 10th percentile were classified as “atypical” (<10th percentile), those in the 10th to 20th percentile range as “borderline,” and those above the 20th percentile as “normal.” Interrater reliability analyses were conducted using the irr package in R (48). All other analyses were conducted using Jamovi 1.6.3 (49).

## Results

### Exploratory Factor Analysis

The Kaiser-Meyer-Olkin (KMO) measure of sampling adequacy was 0.810 for CL and 0.801 for CW. Bartlett's test of sphericity was significant (*p* < 0.001) for both CL and CW. Results of the EFAs are reported in Table 4. Based on parallel analysis, EFA yielded one factor both for CL (eigen-value = 12.70, 64% explained variance) and for CW (eigen-value = 9.30, 47% explained variance). The factor loadings for CL were relatively high, ranging from 0.50 to 0.94 (median loading = 0.83). For CW, loadings were somewhat lower, ranging from 0.39 to 0.80 (median loading = 0.71). Notably, item 10 (“gives irrelevant information”) is reversed-scaled, and should thus give a negative loading. However, for CL and CW item 10 loaded positively on the factor, which indicates that, contrary to expectation, item 10 is not related to SC. Notably, the one factor solution indicates that the QSC-ID does not differentiate between the aspects of SC such as engagement in SC, conversational skills, or pro-social use of communication (see section Construction of the Instrument), that were considered in the development.

**Table 4:** Internal consistency QSC-ID.

	***CL***				***CW***			
	**M**	***SD***	***r_***it***_***	**λ**	***M***	***SD***	***r_***it***_***	**Λ**
1. Starts conversation	4.07	1.072	0.802	0.833	3.92	1.105	0.492	0.542
2. Talks w peers	3.22	1.219	0.782	0.780	3.17	1.298	0.719	0.762
3. Talks w caregivers	4.11	0.847	0.533	0.568	3.94	1.040	0.583	0.636
4. Talks w strangers	2.52	1.503	0.695	0.709	2.47	1.253	0.603	0.617
5. Talks w different peers	3.07	1.269	0.841	0.835	2.64	1.150	0.700	0.710
6. Extended conversation	3.04	1.531	0.848	0.851	2.56	1.362	0.756	0.776
7. Conversational rules	3.11	1.368	0.712	0.737	2.75	1.461	0.617	0.630
8. Responds to contributions	2.74	1.375	0.922	0.941	2.44	1.229	0.766	0.799
9. Stays on topic	2.74	1.259	0.813	0.830	2.83	1.207	0.684	0.709
10. Gives irrelevant information^c^	2.89	1.340	−0.493	0.501	3.58	1.228	−0.380	0.388
11. Asks for clarification	2.74	1.289	0.742	0.768	2.25	1.131	0.621	0.651
12. Balanced conversation	2.85	1.406	0.892	0.896	2.33	1.171	0.697	0.712
13. Participates in group conversations	3.04	1.454	0.899	0.918	2.81	1.451	0.744	0.764
14. Involves him/herself in conversations	2.93	1.492	0.905	0.930	2.64	1.313	0.731	0.748
15. Asks in group	2.44	1.476	0.858	0.858	1.75	1.105	0.593	0.601
16. Expressing him/herself in groups	2.67	1.519	0.816	0.839	2.47	1.341	0.720	0.743
17. Compliments and appreciation	2.30	1.295	0.857	0.851	1.83	0.845	0.649	0.647
18. Offers assistance	2.04	1.344	0.750	0.740	1.69	0.920	0.577	0.575
19. Non-verbal communication	3.37	1.079	0.667	0.690	3.00	1.265	0.725	0.749
20. Sense of humor	3.63	1.182	0.687	0.711	3.19	1.369	0.709	0.743
Total score	2.98	1.00	0.959^a^	12.70^b^ (63.6%)	2.71	0.80	0.930^a^	9.30^b^ (46.5%)

*rit, item-scale correlation; λ, factor loading*.

a *Cronbach's alpha*.

b *Eigen-value*.

c *Reversed-scale item*.

### Internal Consistency

As the EFA results suggested that a single factor underlies the items, we estimated Cronbach's alpha and item-scale correlations *r*_*it*_ over all items. Cronbach's alpha was excellent for CL (0.96) and CW (0.93). In line with the EFA results, the item-scale correlations for the reversed-scale item 10 were negative (item 10 was recoded for reliability analyses). All other items showed moderate to high item-scale correlations (0.53 ≤ *r*_*it*_ ≤ 0.92, median *r*_*it*_ = 0.81 for CL and 0.49 ≤ *r*_*it*_ ≤ 0.77, median *r*_*it*_ = 0.70 for CW). After excluding item 10, internal consistency improved somewhat (CL: Cronbach's alpha = 0.97, 0.55 ≤ *r*_*it*_ ≤ 0.93, median *r*_*it*_ = 0.82; CW: Cronbach's alpha = 0.94, 0.51 ≤ *r*_*it*_ ≤ 0.77, median *r*_*it*_ = 0.71). Moreover, due to high alpha values, we searched for highly correlated items that might indicate redundancies in the item content. We found that items 13 (participates in group conversations) and 14 (involves him/herself in conversations) were highly correlated for CW (*r* =0.95, *p* < 0.001) and CL (*r* = 0.81, *p* < 0.001). For further analysis, we excluded item 10 and computed a scale score by averaging the remaining 19 items separately for CW and CL. Items 13 and 14 were provisionally retained but should be considered in the further development of the QSC-ID.

**Figure 2:**
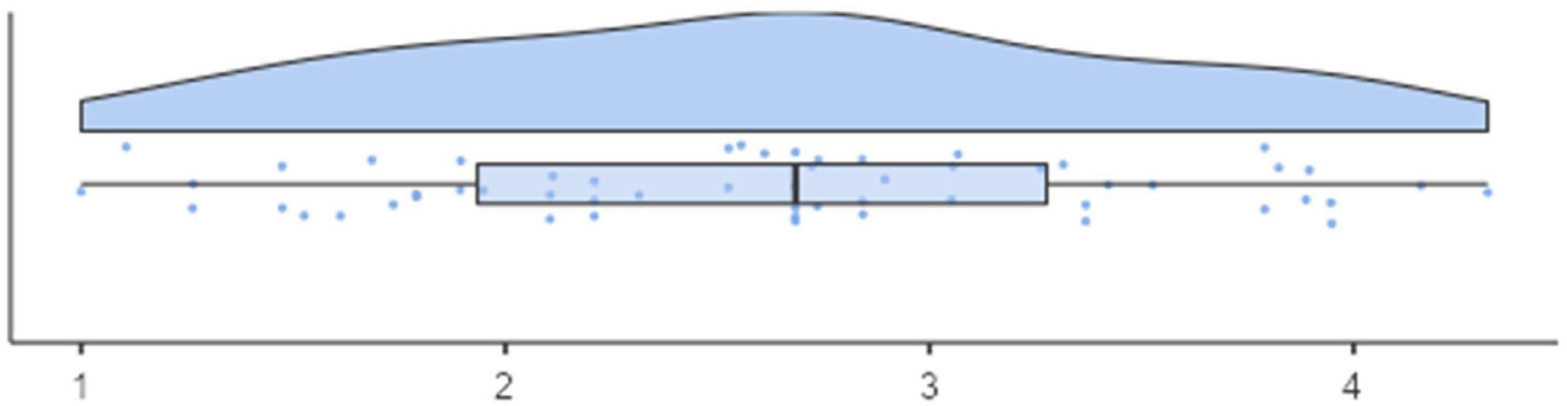
Distribution of QSC-ID results (*M* = 2.74, *SD* =0.83) for participants in the working facilities.

**Figure 3:**
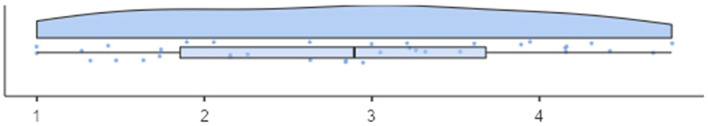
Distribution of QSC-ID results (*MD* = 2.93, *SD* = 1.07) for participants in the living facilities.

Figures 2, 3 present the distributions of the scale scores. The scale scores are not skewed (skewness < |0.04|), hardly kurtotic (kurtosis < |1.01|), and do not deviate from normality (Shapiro-Wilk *W* = 0.979/0.968, *p*s > 0.05). Furthermore, their distribution covers almost the entire theoretical range of values (min = 1, max = 4.79 for CL and 4.32 for CW).

### Interrater Reliability

Table 5 gives the results of the interrater reliability analysis. In addition to ICCs for single items and the total scale score, we also report mean differences (Cohen's *d*) between CL and CW. For the sake of completeness, we also show the results for item 10, which was excluded from the scale score calculation. ICC (0.80) for the total score indicates good interrater reliability. At the item level, interrater reliability was largely moderate, and poor for items 10, 15, and 17 (ICC <0.50). Total scale scores were slightly higher for CW, but the difference was not significant (Cohen's *d* = 0.157, *p* > 0.05). Mean differences at the item level were largely negligible. However, for item 3 (“communicates with caregivers”) the difference was significant, albeit small (Cohen's *d* = 0.384, *p* < 0.05).

**Table 5:** Social communication scores in living and working environment and interrater reliability.

		**CL**		**CW**		**Difference**		**Interrater reliability**	
	***n***	***M***	***SD***	***M***	***SD***	**Effect size**	***p***	***ICC***	**95%-CI**
1. Starts conversation	32	4.03	1.150	3.84	1.081	0.156	0.385	0.590	(0.160, 0.800)
2. Talks w peers	34	3.21	1.225	3.00	1.101	0.184	0.292	0.698	(0.395, 0.849)
3. Talks w caregivers	34	4.15	0.989	3.82	0.936	0.384	0.032	0.763	(0.526, 0.882)
4. Talks w strangers	32	2.38	1.338	2.31	1.030	0.047	0.790	0.562	(0.102, 0.786)
5. Talks w different peers	33	2.97	1.212	2.61	1.059	0.319	0.076	0.664	(0.320, 0.834)
6. Extended conversation	32	3.06	1.544	2.69	1.401	0.280	0.123	0.741	(0.469, 0.873)
7. Conversational rules	32	3.06	1.268	3.06	1.318	0.000	1.000	0.513	(0.001, 0.762)
8. Responds to contributions	33	2.64	1.365	2.70	1.159	−0.056	0.751	0.774	(0.542, 0.888)
9. Stays on topic	34	2.79	1.274	3.06	1.071	−0.210	0.230	0.596	(0.191, 0.798)
10. Gives irrelevant information^c^	32	2.97	1.332	2.75	1.078	0.146	0.415	0.382	(−0.265, 0.698)
11. Asks for clarification	34	2.76	1.327	2.56	1.133	0.165	0.344	0.655	(0.309, 0.828)
12. Balanced conversation	32	2.78	1.338	2.59	0.979	0.172	0.338	0.724	(0.434, 0.865)
13. Participates in group conversations	30	2.83	1.416	2.97	1.426	−0.084	0.650	0.543	(0.004, 0.783)
14. Involves him/herself in conversations	30	2.97	1.426	2.77	1.251	0.130	0.483	0.508	(−0.033, 0.766)
15. Asks in group	28	2.43	1.399	1.93	1.086	0.338	0.085	0.466	(−0.155, 0.753)
16. Expressing him/herself in groups	32	2.69	1.491	2.63	1.338	0.042	0.813	0.652	(0.231, 0.817)
17. Compliments and appreciation	33	2.36	1.245	2.24	1.062	0.090	0.607	0.495	(−0.023, 0.751)
18. Offers assistance	33	1.97	1.262	1.76	0.902	0.191	0.281	0.655	(0.302, 0.830)
19. Non-verbal communication	34	3.26	1.109	3.38	1.129	−0.108	0.535	0.686	(0.371, 0.843)
20. Sense of humor	33	3.45	1.277	3.42	1.251	0.024	0.891	0.668	(0.328, 0.836)
Total score	34	2.71	1.066	2.82	0.773	0.157	0.367	0.803	(0.605, 0.902)

c *Reversed-scale item*.

### Convergent and Discriminant Validity

Table 6 presents correlations between QSC-ID raw scores and the results of measures of language, social skills, adaptive skills, non-verbal intelligence, and motor skills.

**Table 6:** Correlates of QSC-ID raw scores as indicators of convergent and discrimination validity.

**RDLS**	**PMLP**	**V-ABS**	**DiBAS-R**	**V-ABS**	**Non-verbal cognition**	**V-ABS**
Language. comprehension (total score)	Expressive language level	Social skills (total score)	Autism symptoms (total score)	Daily living skills (total score)	(Reference age)	Motor skills (total score)
0.543***	0.737***	0.504***	−0.689***	0.344*	0.489***	0.243

*CDI, child development inventory; RDLS, Reynell developmental language scale; PMLP, profile of multiple language proficiencies (adapted to Austrian sign language); V-ABS, Vineland adaptive behavior scales; DiBAS, diagnostic observational sheet for autism spectrum disorders; domains of development are marked according to their expected correlations to SC in green (strong correlation), yellow (moderate correlation), or red (weak or nor correlation)*.

* *p < 0.05*,

*** *p < 0.001*.

The results largely confirmed our expectations (Table 6). The correlations between QSC-ID total scores and language, social and ASD scores were high, moderate for adaptive skills, and non-verbal cognition and non-significant for motor skills.

### Feasibility

Caregiver feedback, response rate, and completeness of data suggest high feasibility of the developed instrument. The QSC-ID questionnaires had a 96.0% response rate (86 out of 90). Eighty-five percent of the tests were fully completed, which suggests good practicability of the instrument. Thirteen questionnaires were incomplete, the majority of which were missing only a single item. In total, no more than four items were omitted per questionnaire. Table 7 gives an overview of the caregiver feedback on feasibility. Completing the QSC-ID took about 10 min, and for the majority of caregivers all questions and examples given in the QSC-ID were easy to understand. Ninety-five percent regarded the questionnaire as helpful in evaluating a client's SC status.

**Table 7:** Feasibility QSC-ID.

**Caregiver feedback**	
Completing the questionnaire took me…	
*Less than 5 min*	4 (20.0)
*Between 5 and 10 min*	9 (45.0)
*Between 10 and 15 min*	5 (25.0)
*Longer than 15 min*	2 (10.0)
Completing the questionnaire was…	
*Easy*	15 (75.0)
*Acceptable*	5 (25.0)
*Difficult*	–
The questions and examples were easy to understand.	
*Fully*	14 (70.0)
*Mostly*	6 (30.0)
*Partly*	–
*Not at all*	–
The questionnaire is useful for evaluating a client's SC status.	
*Not at all*	–
*A little*	1 (10.0)
*Moderately*	13 (65.0)
*Very*	6 (30.0)
Response rate	86 (96.0)
Completeness of data	73 (85.0)

### Calculation of Cut-Off Points

The “atypical” group with percentile scores below 10 is characterized by raw scores of ≤ 29 and symptoms of severe SC deficits relative to the remaining sample. In the current sample four individuals were classified as “atypical.” All of them were male and had a diagnosis of ASD. Of the total sample (*n* = 52) seven had clinical diagnoses of ASD. Levels of non-verbal cognitive functioning varied between 4.05 and 6.03 years. All of them showed severe language delays with an average reference age of 2.06 years in language comprehension and an average PMLP expressive language level of 2.88, which is between the level of first word combinations and the ability to combine isolated signs to create simple questions, stereotypical phrases, and expressions. Non-verbal cognitive, adaptive, and language skills were very similar to the borderline group but significantly below the scores of the participants classified as “normal” (Table 8).

**Table 8:** Characteristics of subgroups classified as “atypical,” “borderline,” and “normal.”

	**Atypical**	**Borderline**	**Normal**	***p*** **-Values^a^**
	** <10%**	**10–20%**	**>20%**	
	***n*** **= 4**	***n*** **= 6**	***n*** **= 42**	
Age *M* (*SD*)	48.25 (19.36)	23.67 (8.62)	48.83 (19.01)	0.036
Sex male *n* (%)	4 (100.0)	5 (84.0)	24 (57.0)	0.052
Non-verbal cognitive functioning				0.067
Reference age *n* (%)				
*>6 years*	1 (33.0)	1 (17.0)	21 (51.0)	
*3–6 years*	2 (67.0)	4 (67.0)	20 (49.0)	
* <3 years*	0 (0.0)	1 (17.0)	0 (0.0)	
RDLS language comprehension	29.50 (0.71)	32.83 (17.18)	40.57 (10.22)	0.152
(Total score) *M* (*SD*)				
PMLP expressive language level *M* (SD)	2.88 (1.44)	3.08 (1.36)	4.91 (1.69)	0.002
*V-ABS adaptive skills (reference age)*	0.51 (0.58)	2.05 (1.67)	5.70 (3.43)	<0.001
Degree of hearing loss^a^ *n* (%)				1.000
*Moderate hearing loss (40–69 dB)*	0 (0.0)	0 (0.0)	1 (2.0)	
*Severe hearing loss (70–89 dB)*	0 (0.0)	1 (17.0)	6 (14.0)	
*Profound hearing loss (>89 dB)*	4 (100.0)	5 (83.0)	35 (83.0)	
Autism spectrum disorder *n* (%)	4 (100.0)	2 (33.0)	1 (3.0)	<0.001
Epilepsy *n* (%)	2 (50.0)	3 (50.0)	8 (19.0)	0.097
Cerebral palsy *n* (%)	0 (0.0)	2 (33.0)	14 (33.0)	0.480

*RDLS, Reynell developmental language scale; PMLP, profile of multiple language proficiencies (adapted to Austrian sign language); V-ABS, Vineland adaptive behavior scales*.

a *p-values for the comparison between normal (>20%) and atypical or borderline (≤ 20%). p-values are based on Fisher's exact tests for categorical variables and on Mann-Whitney U tests for continuous variables*.

Participants of the “borderline SC” group (10–20%) with total raw scores from 30 to 36 (*n* = 6) were characterized by severe developmental delays of non-verbal cognitive (with scores for reference age under 3.0 years in four of them) and adaptive functioning and severe language delays very similar to the “atypical” subsample. The “borderline” group included one male and one female participant with ASD characterized by similar total QSC-ID scores (f 31.5 and m 33), but marked differences in their non-verbal cognitive functioning age (f 4.01 years and m 6.01 years) and RDLS receptive language profiles (f 2.04 years and m 4.06 years) demonstrating that the SC construct measured by the QSC-ID is different from non-verbal cognition or language skills. What distinguished them from the four participants with ASD and atypical SC was that, depending on setting, topic, and interlocutor, both of them showed some adequate SC behaviors. The female participant enjoyed communication in particular with her key caregivers, even if mainly non-verbally. If things were important to her, she showed clear communication initiatives and could express her needs communicatively. The male participant showed communicative initiatives most of the time and he enjoyed to communicate with caregivers as well as with peers. Compared to the female participant, he often imitated the behavior of others and had more difficulties to get into a two-way communication.

Participants with a QSC-ID raw score above 36 were classified as “normal”. As described above, their average level of cognitive, adaptive as well as linguistic functioning was significantly above that of the “atypical” and “borderline” groups. There were no statistically significant differences for degree of hearing loss or the percentage of individuals with cerebral palsy or epilepsy. A number of participants with severe ID and low language skills in the group with typical SC results confirmed that low levels of non-verbal cognitive functioning and severely restricted language are not automatically correlated with low SC total scores. They usually showed high initiative in communication by use of vocalizations, pointing, showing things and simple gestures or non-conventional signs, good eye contact, simple reciprocity, and came across as thoroughly socially motivated. There was just one participant in the group classified as typical (QSC-ID total score = 39.5) with regard to SC where we had anticipated lower SC results. This young man with one of the highest non-verbal intelligence quotient in the sample showed difficulties in initiating a conversation on his own, nevertheless he enjoyed communication with caregivers and other clients. Conversations with him tended to be short and restricted to everyday topics but still balanced. In terms of simple repair strategies the participant commonly expressed difficulties in understanding signed communication. He managed quite well to adhere to conversational rules and to stay on topic. The participant's questionnaire, however, revealed clear weaknesses in pro-SC skills (e.g., praise, compliments) and in the ability to attribute mental states to his interlocutors.

## Discussion

Our results suggest that the QSC-ID might fill a gap in the field of intervention and research into communication difficulties in adults with ID. Construct validity of the questionnaire was found to be high. As expected, correlations with language, social skills, and with symptoms of ASD were high, moderate for adaptive skills and non-verbal intelligence, and non-significant for motor-skills.

Total raw scores were well-distributed over the whole range. Interrater reliability was found to be good or at least acceptable for all items. There were slight but insignificant differences between the ratings of caregivers in the living facility (CL) and those in the workshops (CW). For the poor ICC of item 3 (“talking with caregivers”), it can be assumed that rater biases were mainly due to the context in which communication happened and was observed by caregivers, as an individual's SC behavior is influenced by social context. Conversational situations and needs are much more diverse during time spent in living facilities than during working time. Time at work is highly structured due to the institution's therapeutic concept, and conversational topics are often ritualized and repetitive, whereas the group living environment offers far more space and opportunities for extended conversations and for addressing a wider variety of topics and communicative needs.

Analyses identified item 10 (“Tells others things they already know or don't care about.”) as a reversed-scale item. It refers to the adaptation of communication to the interlocutor, which requires the ability to attribute mental states such as knowledge, intentions, or belief, an important skill in the context of SC. For this item to be readmitted to the questionnaire, its wording would have to be adjusted and revalidated. Very high correlations between items 13 and 14 (participating vs. involving oneself in group conversations) suggest that the difference between attendance and active contribution to group conversations needs to be clarified in future developments of the questionnaire's items.

Caregiver feedback, response rate, and completeness of data show high feasibility of the questionnaire (particularly regarding acceptance, time economy, understanding, and usefulness of the QSC-ID).

Six out of seven participants with clinical diagnoses of ASD were classified by the QSC-ID as “atypical SC” (*n* = 4, PR < 10th) or “borderline SC” (*n* = 2, PR 10th-20th), and all of the individuals classified as “atypical” had a diagnosis of ASD. Particularly, conversational interaction and non-verbal communication items of the questionnaire were abnormal in adults with ID and a known diagnosis of ASD. Results suggest, that the new questionnaire might assist in the identification of ASD. However, as the QSC was not designed as an ASD screener other core characteristics of ASD (restrictive and repetitive behaviors or sensory reactivity) are not included. Over and above, the group with borderline SC scores showed that SC deficits were not necessarily connected with ASD.

Since the QSC-ID scores were not skewed and did not deviate from normality the questionnaire might be helpful not only for the detection of SC problems but also useful for the identification of strengths that need to be integrated into intervention planning.

The participants in our pilot study represent a very specific and heterogeneous population for whom there is no standard sample. Even though the current sample represents the largest cohort of adults with ID and deafness published in the literature to date, a larger population would be needed for more accurate validation of the questionnaire. Further, only cross-sectional data are currently available, and no conclusive statements can be made regarding sensitivity to change and the measurement of developmental progressions. In addition, validation of the questionnaire in populations with ID and typical hearing including children and adolescents is required. With increasing data the calculation of specific cut-offs for abnormal or borderline SC skills for different levels of linguistic skills should be considered as a high correlation between SC scores and structural language skills was found in the pilot study. Similarly, the Children's Communication Checklist-2 (50) provides an index (social interaction difference index) to discriminate between pragmatic problems as a consequence of limited structural language and those that are significantly more pronounced than the language difficulties. The use of double-barreled items in the questionnaire can be regarded as another limitation of the new measure as they might have caused irritation among the care-givers.

## Conclusion

The results of the pilot study indicate that the QSC-ID is a valid single-factorial instrument for the identification of SC deficits and strengths in individuals with ID. It is an easy-to-understand questionnaire that can be completed within about 10 min. The items of the QSC-ID have been formulated to be independent of communication mode, and the new measurement works well with individuals with mild to severe ID. Social communication problems identified by the QSC-ID, particularly in case of high severity, are often linked with ASD. However, SC deficits are not necessarily combined with ASD and can be distinguished from non-verbal cognitive and linguistic deficits. The QSC-ID should be further tested in clinical practice of multi-dimensional assessment in individuals with ID. At the moment, the suggested SC cut-offs need to be used with caution, and interpretation of the SC score with respect to the individual's language level is recommended. In addition to clinical trials, inclusion of the QSC-ID in research is recommend to explore the correlations of SC with other developmental areas, such as social development, language development, social cognition and adaptive skills, and for investigating the role of SC skills as correlate and predictor of mental health and quality of life in people with ID. Since SC scores have been found to be highly correlated with language skills development of a discrepancy criterion between SC and structural language skills should be considered.

## Data Availability Statement

The datasets presented in this article are not readily available because legal guardians and participants have not been asked for permission to share the original data. Requests to access the datasets should be directed to daniel.holzinger@bblinz.at.

## Ethics Statement

The studies involving human participants were reviewed and approved by Ethikkommission Barmherzige Schwestern und Barmherzige Brüder. The patients/participants provided their written informed consent to participate in this study.

## Author Contributions

DH, JF, and ChaW: design of the study and first draft of document. ChaW: data collection. ChrW: statistical analysis. All authors finally reviewed the manuscript.

## Funding

Article Processing Charge was funded by the Johannes Kepler University Open Access Publishing Fund.

## Conflict of Interest

The authors declare that the research was conducted in the absence of any commercial or financial relationships that could be construed as a potential conflict of interest.

## Publisher's Note

All claims expressed in this article are solely those of the authors and do not necessarily represent those of their affiliated organizations, or those of the publisher, the editors and the reviewers. Any product that may be evaluated in this article, or claim that may be made by its manufacturer, is not guaranteed or endorsed by the publisher.
